# Supplementary figures and images for: Incidence of malignant transformation in the oviductal fimbria in laying hens, a preclinical model of spontaneous ovarian cancer
Source: PLoS One. 2021 Jul 27;16(7):e0255007. doi: 10.1371/journal.pone.0255007 (PMC8315513; doi:10.1371/journal.pone.0255007)

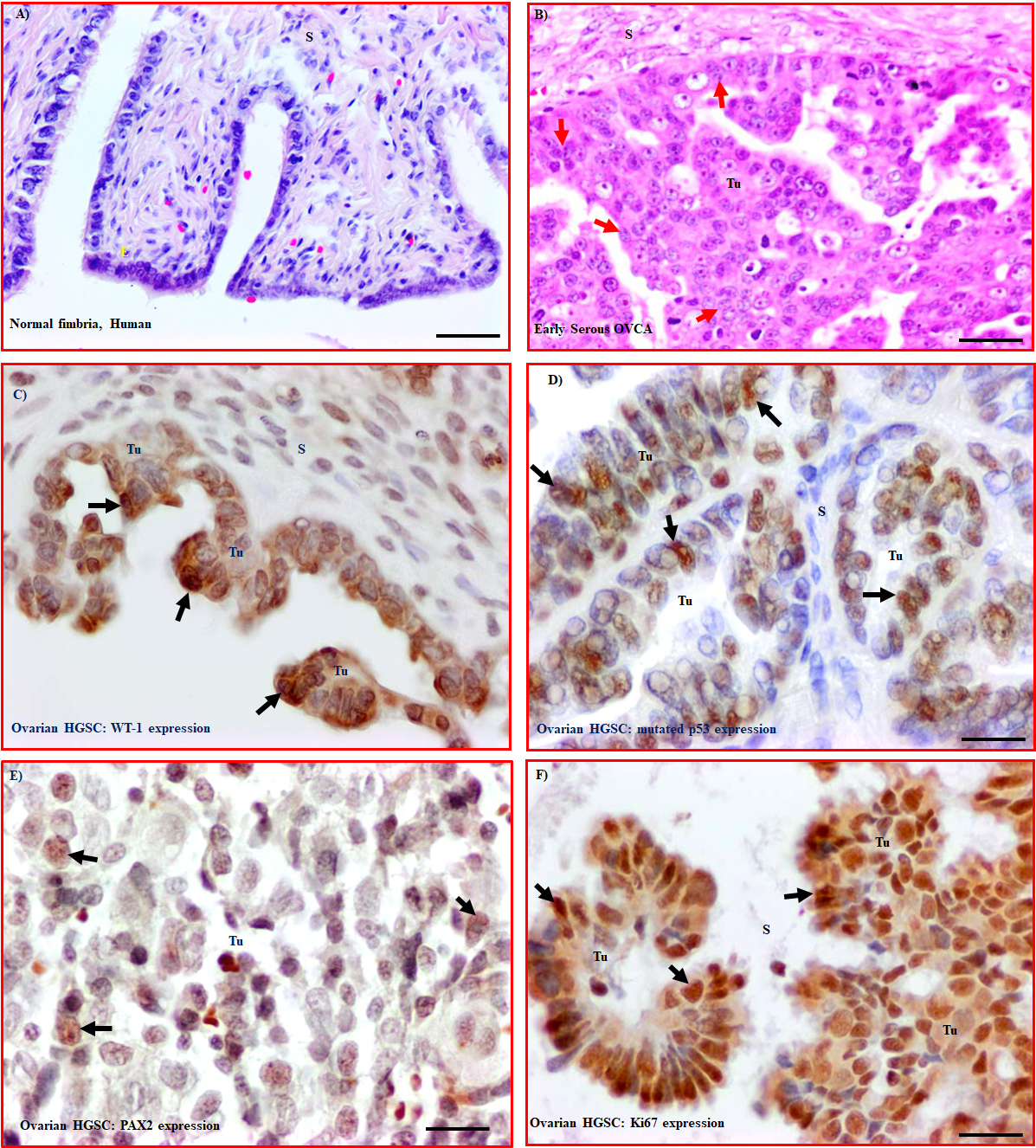

Supplement: S1 Fig — (A) Section of a normal fimbria from a subject. Histological features are similar with the fimbria in hens shown in Fig 2. (B) Section of an ovarian HGSC showing papillary-like feature of the tumor surrounded by stroma. Such features were also seen in fimbria with tumor in hens (Fig 2). (C-F) Sections of ovarian HGSC stained for expression of WT-1, mutated p53, PAX2 and Ki67 staining, respectively. Similar patterns of staining for these markers were also observed in fimbriae with tumor in hens (presented in Figs 3–5). E = Ovarian surface epithelial cells, S = Stroma, Tu = Tumor. Scale bar = 20μm. (TIF) [file pone.0255007.s001.tif]

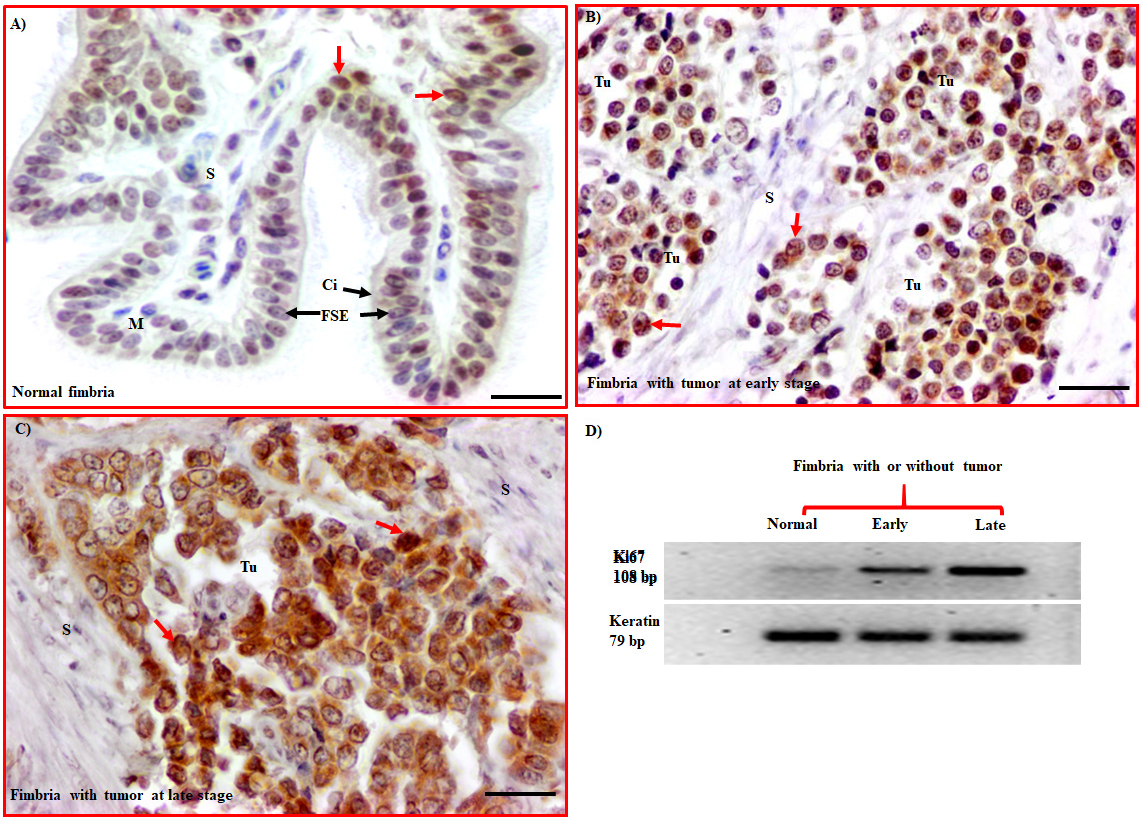

Supplement: S2 Fig — (A) Surface epithelial cells in normal fimbria (FSE) showed variable staining including strong staining by apical cells while basal cells showed weak staining for Ki67 expression. (B and C) In contrast to normal fimbria, intense staining for Ki67 staining was observed in fimbriae with tumor at early stage (B) and late stage (C). D) RT-PCR showing gene expression of Ki67 and keratin 8 was used as control. As in immunohistochemistry, strong signal for Ki67 in fimbriae with tumor was also observed in gene expression assay. Ci = Cilia, M = Mucosa of the fimbria, S = Stroma, Tu = Tumor. Scale bar = 20μm. (TIF) [file pone.0255007.s002.tif]
