# Supplementary material for: Incidence of malignant transformation in the oviductal fimbria in laying hens, a preclinical model of spontaneous ovarian cancer
Source: PLoS One. 2021 Jul 27;16(7):e0255007. doi: 10.1371/journal.pone.0255007 (PMC8315513; doi:10.1371/journal.pone.0255007)
Supplement: S1 Table — (DOCX) [file pone.0255007.s003.docx]

**S1 Table. Information on primary antibodies used in this study.**

| **Antibody** | **Catalog Number** | **Lot Number** | **Host** | **Company** |
| --- | --- | --- | --- | --- |
| Anti-WT-1 | SAB2108752 | QC7087 | Rabbit | Millipore Sigma |
| Anti-p53 | OP29L-100UG | 3275117 | Mouse | Millipore Sigma |
| Anti-Ki67 | AB9260 | 2886469 | Rabbit | Millipore Sigma |
| Anti-CA-125 | 187260 | 785184A | Mouse | Thermo Fisher Scientific |
| Anti-PAX2 | AF3364 | XOT0320121 | Goat | R&D Systems, Inc. |
