# Supplementary material for: Incidence of malignant transformation in the oviductal fimbria in laying hens, a preclinical model of spontaneous ovarian cancer
Source: PLoS One. 2021 Jul 27;16(7):e0255007. doi: 10.1371/journal.pone.0255007 (PMC8315513; doi:10.1371/journal.pone.0255007)
Supplement: S2 Table — (DOCX) [file pone.0255007.s004.docx]

**S2 Table. Distribution of hens with or without ovarian tumors based on their gross presentation.**

| Groups of hens | Gross presentation | Number of cases (hens) |
| --- | --- | --- |
| Group-1 | Healthy hens without any abnormality | 105 |
| Group-2 | Solid masses in the fimbria and/or other parts of the infundibulum of the oviduct as well as in a part of the ovary | 7 |
| Group-3 | Solid mass only in the ovary without the involvement of the fimbria of the oviduct | 8 |
| Group-4 | Solid masses in the ovary, oviduct and metastasized to other distant organs | 12 |
