# Supplementary material for: Incidence of malignant transformation in the oviductal fimbria in laying hens, a preclinical model of spontaneous ovarian cancer
Source: PLoS One. 2021 Jul 27;16(7):e0255007. doi: 10.1371/journal.pone.0255007 (PMC8315513; doi:10.1371/journal.pone.0255007)

S1 Raw Images. Original blot/gel image data used in this study.

Fig 3E (Chemiluminescence)

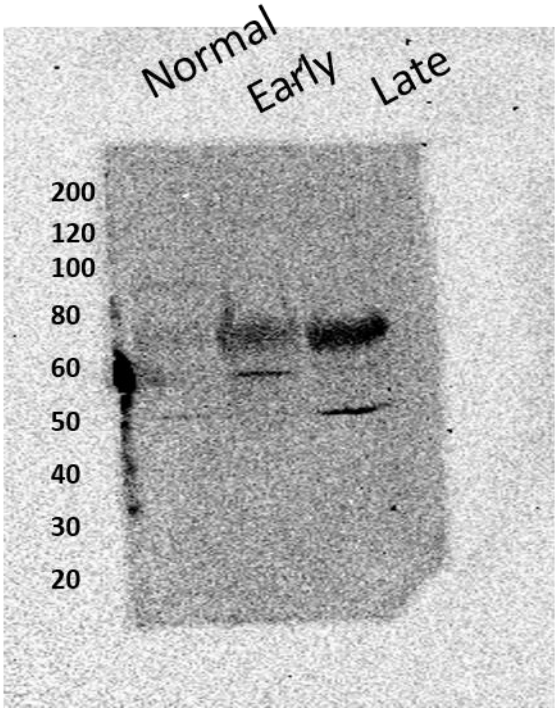

Fig 3F (UV)

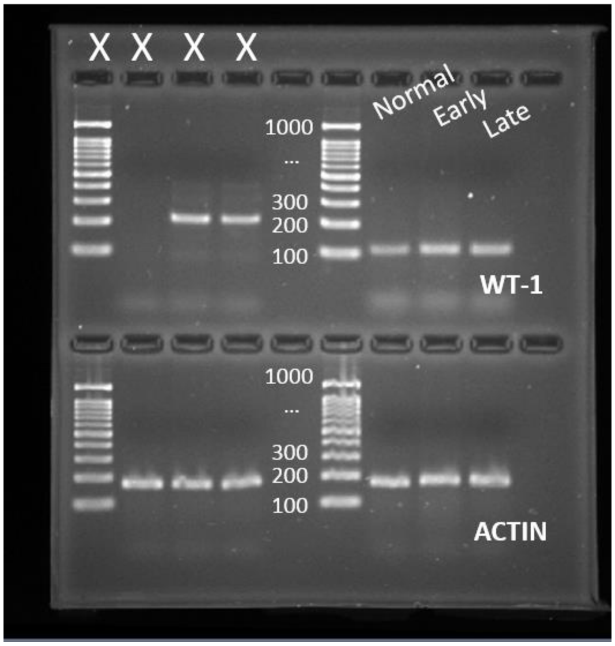

Fig 4D (UV)

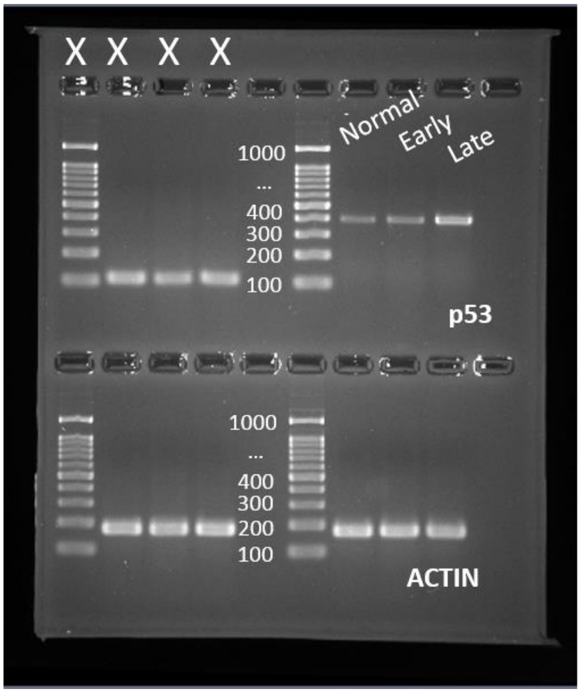

Fig 5A (Chemiluminescence)

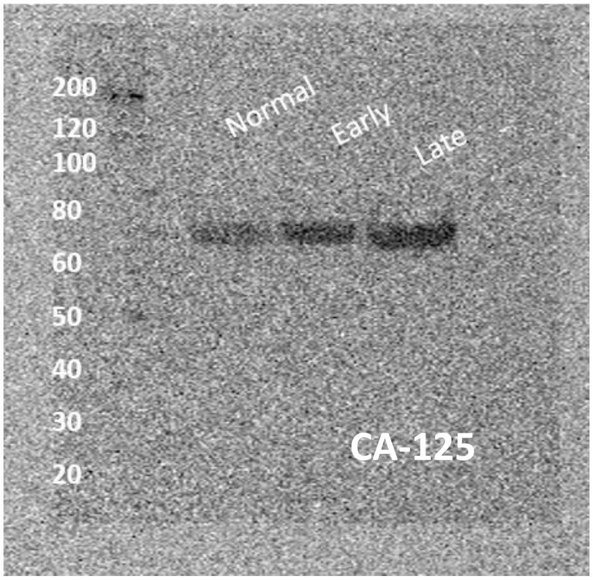

Fig 6E (UV)

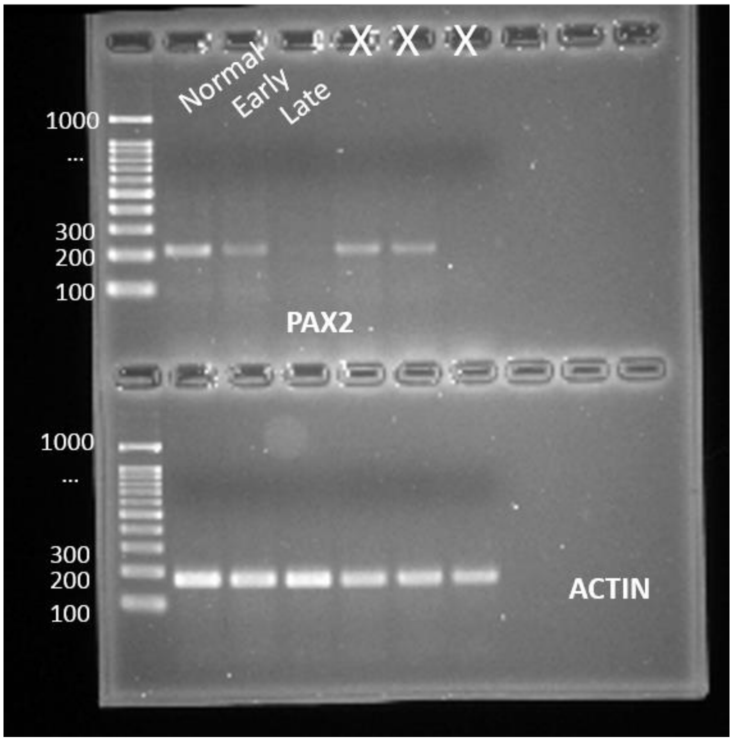

S2Fig D (UV, inverted colors)

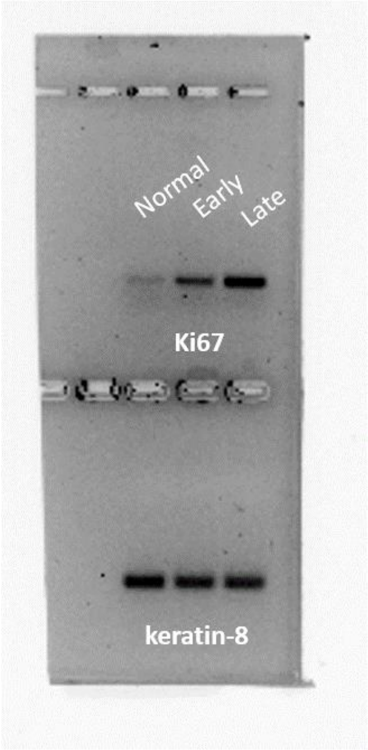

Supplement: S1 Raw images — (PDF) [file pone.0255007.s005.pdf]
